# Analysis of huanglongbing-associated RNA-seq data reveals disturbances in biological processes within *Citrus* spp. triggered by *Candidatus* Liberibacter asiaticus infection

**DOI:** 10.3389/fpls.2024.1388163

**Published:** 2024-04-10

**Authors:** Ruimin Li, Xinyou Wang, Yanan Hu, Guiyan Huang

**Affiliations:** ^1^ College of Life Sciences, Gannan Normal University, Ganzhou, China; ^2^ China-USA Citrus Huanglongbing Joint Laboratory, National Navel Orange Engineering Research Center, Gannan Normal University, Ganzhou, China

**Keywords:** Citrus sinensis, Candidatus Liberibacter asiaticus, RNA-Seq, WGCNA, biological processes

## Abstract

**Introduction:**

Huanglongbing (HLB), a disease that’s ubiquitous worldwide, wreaks havoc on the citrus industry. The primary culprit of HLB is the gram-negative bacterium *Candidatus* Liberibacter asiaticus (CLas) that infects the phloem, but its damaging mechanism is yet to be fully understood.

**Methods and results:**

In this study, a multitude of tools including weighted correlation network analysis (WGCNA), protein-protein interaction (PPI) network analysis and gene expression profiling are employed to unravel the intricacies of its pathogenesis. The investigation pinpoints various central genes, such as the ethylene-responsive transcription factor 9 (*ERF9*) and thioredoxin reductase 1 (*TrxR1*), that are associated with CLas invasion and resultant disturbances in numerous biological operations. Additionally, the study uncovers a range of responses through the detection of differential expressed genes (DEGs) across different experiments. The discovery of core DEGs leads to the identification of pivotal genes such as the sieve element occlusion (*SEO*) and the wall-associated receptor kinase-like 15 (*WAKL15*). PPI network analysis highlights potential vital proteins, while GO and KEGG pathway enrichment analysis illustrate a significant impact on multiple defensive and metabolic pathways. Gene set enrichment analysis (GSEA) indicates significant alterations in biological processes such as leaf senescence and response to biotic stimuli.

**Discussion:**

This all-encompassing approach extends valuable understanding into the pathogenesis of CLas, potentially aiding future research and therapeutic strategies for HLB.

## 1 Introduction

The citrus industry is significantly impacted by citrus huanglongbing (HLB), which is a disease that has spread globally and causes severe damage (Wang, 2019). HLB can affect all citrus varieties, leading to symptoms such as yellowing of leaves, stunted growth, and the production of small, misshapen, and bitter fruits (Bové and Barros, 2006; Wang, 2019; Pandey et al., 2022). Ultimately, the disease leads to premature tree death, rendering orchards unproductive, resulting in many orchards being abandoned or replaced with alternative crops (Yuan et al., 2021).

HLB is typically believed to be caused by *Candidatus* Liberibacter asiaticus (CLas), which is a gram-negative bacterium that parasitizes to the phloem (Zheng et al., 2023). The pathogenesis of CLas is unclear during its unculturable *in vitro* (Hu et al., 2021). Nevertheless, there are still numerous research findings attempting to uncover the pathogenesis of CLas (Pitino et al., 2016; Loto et al., 2017; Clark et al., 2018; Pitino et al., 2018; Shi et al., 2019; Liu et al., 2019a; Du et al., 2021; Wang et al., 2023; Shi et al., 2023a, Shi et al., 2023b). Following the release of the CLas genome, researchers found that the bacterium lacks type III or IV secretory systems, but it does have a complete sec-dependent system (Duan et al., 2009). The Sec-dependent effectors (SDEs) of CLas disrupt autophagy, development, and papain-like cysteine proteases, while also suppressing the immune response in citrus (Clark et al., 2018; Wang et al., 2023; Shi et al., 2023a, Shi et al., 2023b). For instance, SDE1 targets citrus proteases and declines defense responses in plants (Clark et al., 2018). Overexpression of SDE1 accelerated senescence related biological processes both in *Arabidopsis thaliana* and *Citrus paradisi* (Clark et al., 2020). Moreover, SDE15 suppresses the immune response to bacterial infection by interacting with citrus CsACD2 (Pang et al., 2020). Interestingly, both SDE3 and SDE4405 interfere with citrus autophagy; SDE3 suppresses it, while SDE4405 stimulates it (Shi et al., 2023a, Shi et al., 2023b). Nevertheless, both SDE3 and SDE4405 exert a negative influence on the citrus defense response (Shi et al., 2023a, Shi et al., 2023b). Although the functions of several SDEs have been analyzed, the pathogenic mechanism of CLas is still unclear. For instance, whether these SDEs work synergistically or independently during the infection process and which effector protein is the key pathogenic factor, remain uncertain. Moreover, the response of citrus to CLas infection and the regulatory mechanisms involved in citrus need to be clarified. Dynamic changes in the signal transduction pathway also require further investigation.

Upon CLas infection, the citrus transcriptome undergoes significant alterations, leading to the up-regulation or down-regulation of numerous genes (Liu et al., 2023). These genes are primarily associated with plant defense responses, photosynthesis, cell wall modification, nutrient transport, and metabolism among others (Hu et al., 2017; Zhao et al., 2019; Liu et al., 2019b; Ribeiro et al., 2022). Several defense-related genes and pathogenesis-related proteins (PRs) are up-regulated in response to CLas infection. For instance, genes encoding PR proteins, such as peroxidase, chitinase, and β-1,3-glucanase, have been observed to increase, indicating an active defense response (Rawat et al., 2015). Additionally, genes related to secondary metabolite biosynthesis, like phytoalexins, are up-regulated, providing further biochemical defense against the pathogen (Fu et al., 2016). CLas infection has been associated with a broad down-regulation of genes related to photosynthesis and carbohydrate metabolism (Martinelli et al., 2012; Liu et al., 2019b, 2023). The down-regulation of these genes likely contributes to the pronounced leaf yellowing observed in CLas-infected trees. Transcriptomic analysis has revealed an up-regulation of genes related to cell-wall modification, such as those encoding pectin methylesterases and pectate lyases (Wang et al., 2016; Liu et al., 2023). These enzymes are involved in the modification and degradation of the cell wall, suggesting an alteration in plant cell wall integrity and structure during CLas infection. Genes involved in nutrient transport, such as those encoding sugar and amino acid transporters, show variable expression patterns depending on the stage of CLas infection (Shahzad et al., 2020). These intricate changes in the citrus transcriptome in response to CLas infection underscore the complexity of the plant-pathogen interaction. Notably, the up-regulation of defense-related genes suggests a robust, yet not fully effective, physiological response to the pathogen, while the down-regulation of photosynthetic genes highlights the detrimental effects of HLB on the primary metabolic processes of citrus. Despite ongoing transcriptomic research on HLB, the conservative response regulation pattern to CLas infection in citrus remains unclear due to the different stages of CLas infection and the variety of citrus species involved. Therefore, in this study, we utilize the previous sequencing data from our research group and publicly available data from National Center for Biotechnology Information (NCBI) SRA database to determine the core regulatory genes, conservative biological response processes, metabolic pathways, and response patterns of citrus.

Weighted gene co-expression network analysis (WGCNA) offers a robust computational approach to decipher complex biological systems at the genetic level (Langfelder and Horvath, 2008). The performance of WGCNA in identifying co-expression modules has become a critical tool in plant transcriptional regulation (Lu et al., 2019; Zhu et al., 2019; Yu et al., 2023). WGCNA is helpful to identify hub genes that control plant growth, development, and responses to environmental stimuli (Yao et al., 2023; Yu et al., 2023). For instance, multiple transcription factors associated with regulation of salt response in rice were identified by WGCNA (Zhu et al., 2019). Moreover, Gene set enrichment analysis (GSEA) is an important computational method in analyzing transcriptomic data, particularly in studies focused on plant-pathogen interactions (Muchero et al., 2018; Rody et al., 2021). Understanding the complex and dynamic interplay between a plant and its pathogen at a molecular level requires tools that can identify and categorize patterns in gene expression (Dong et al., 2015; Jiang et al., 2017). Applications of GSEA in plant-pathogen interaction studies have led to the identification of key gene sets and pathways that are modulated during infection (Bautista et al., 2021). Furthermore, the utilization of Gene ontology (GO) and Kyoto Encyclopedia of genes and genomes (KEGG) enrichment analyses enables identification of the biological processes and metabolic pathways in which differentially expressed genes (DEGs) are involved (Liu et al., 2023).

In order to elucidate the disturbances in biological processes within *Citrus* spp. triggered by CLas infection, we collected HLB associated RNA-seq data, including the RNA-seq data sequenced in our previous study (Liu et al., 2023) and public RNA-seq data deposited in NCBI SRA database from various research groups, for analysis. Subsequently, we utilized WGCNA to identify gene sets and pinpointed hub genes positive correlated with CLas infection. Moreover, we performed GO and KEGG enrichment analysis, protein-protein interactions (PPI) network analysis and GESA to determine biological processes and metabolic pathways disturbed by CLas infection. Our findings would lay the groundwork for better understanding of CLas pathogenesis, elucidating the complex interaction and molecular mechanisms between citrus and CLas.

## 2 Materials and methods

### 2.1 RNA-seq data collection and analysis

To collect HLB associated RNA-seq data, we used ‘*Citrus*’, ‘*Candidatus* Liberibacter asiaticus’, and ‘Huanglongbing’ as keywords to search related biosamples in SRA database. In addition, we incorporated RNA-seq data from a previous publication by our research group into this study (Liu et al., 2023). We then utilized the ‘Kallisto Super GUI Wrapper’ within the TBtools software to quantify the expression profiles of genes in *C. sinensis* using the 3.0 version reference genome in the Citrus Pan-genome to Breeding Database (CPBD) (http://citrus.hzau.edu.cn/) (Bray et al., 2016; Liu et al., 2022; Chen et al., 2023). The bias correction parameters were configured with a kmer size of 31.

### 2.2 Weighted correlation network analysis

The WGCNA was performed with WGCNA-shinyApp (https://github.com/ShawnWx2019/WGCNA-shinyApp) (Langfelder and Horvath, 2008). The raw count values of all genes were normalized using the variance-stabilizing transformation method (Lin et al., 2008), and then the gene sets were filtered twice. First, genes with 90% of samples having a count value less than 10 were removed. Then, genes were further filtered using the ‘median absolute deviation’ method (Pham-Gia and Hung, 2001). The normalized count values of the remaining genes were utilized to calculate the suggested power value. Then module net was constructed with parameters of ‘min Module size = 30’ and ‘module cuttree height = 0.25’. All CLas infected samples were designated as ‘HLB’, while CLas free samples were designated as ‘MOCK’. The correlation between module and trait data (such as ‘HLB’) was computed, and significant ‘module-trait’ pairs were utilized to identify hub genes.

### 2.3 Hub genes identification

The selection of hub genes was determined by filtering with a cut off value greater than 0.5 for both the ‘kME’ and ‘GS’ generated with WGCNA-shinyApp. Subsequently, the edge information for each module was generated and any weight values below 0.8 were eliminated. We then utilized Cytoscape 3.9.0 to arrange the co-expression network (Kohl et al., 2011).

### 2.4 Differential expressed genes identification

DEGs were generated with R package ‘edgeR’, with a threshold of absolute log fold change (|log2FC|) greater than 1 and a false discovery rate of less than 0.05 (Robinson et al., 2010). The DEGs from various experiments were combined, and those found in over 2/3 of the experiments were classified as core DEGs (cDEGs), while those found in more than 1/2 of the experiments were classified as soft core DEGs (scDEGs). The Venn diagram was drawn by InteractiVenn (Heberle et al., 2015), and the heatmap was drawn by TBtools (Chen et al., 2023), while the GO IDs were clustered by simplifyEnrichment (Gu and Hübschmann, 2023).

### 2.5 Protein-protein interaction network analysis

cDEGs were utilized for conducting PPI network analysis with the STRING database (Szklarczyk et al., 2019), and subsequently, the network was visualized using OmicSuite software (Miao et al., 2023). The annotation of proteins in the network was verified through blastp in the NCBI database (Johnson et al., 2008).

### 2.6 GO and KEGG pathway enrichment analysis

scDEGs were used for GO and KEGG pathway enrichment analysis. The log2FC value for each scDEG was calculated by taking the log 2 of the average count value of all CLas infected samples dividing the average count value of all CLas free samples. The GO terms of all genes in *C. sinensis* were annotated by eggNOG-mapper 2.1.12 (Cantalapiedra et al., 2021), while the KEGG ID of each gene of *C. sinensis* were annotated by KofamKOALA (Aramaki et al., 2020). Enrichment analysis for both GO and KEGG pathways was carried out using gogseasenior and pathwaygseasenior online tools on the omicshare website (www.omicshare.com).

### 2.7 Gene set enrichment analysis

GSEA was conducted using ‘Simple GO GSEA Wrapper’ within the TBtools software (Subramanian et al., 2005; Chen et al., 2023). scDEGs were used for gene ranking and gene sets with *P* value less than 0.05 were considered as significantly enriched gene sets.

### 2.8 qRT-PCR analysis

Quantitative real-time PCR (qRT-PCR) was implemented to corroborate the RNA-seq data. We gathered mature leaves from both CLas-infected and CLas-free *C. sinensis* cv. “Newhall”, from which total RNA was extracted utilizing the *EasyPure*® Plant RNA Kit (Transgen Biotech, Beijing). RNA was subsequently reverse-transcribed to produce first-strand cDNA by means of the *EasyScript*® All-in-One First-Strand cDNA Synthesis SuperMix for qPCR (One-Step gDNA Removal) (Transgen Biotech, Beijing). Six arbitrarily chosen DEGs up-regulated in CLas-infected samples were subjected to qRT-PCR analysis with *TransStart*® Green qPCR SuperMix (Transgen Biotech, Beijing) employing specific primers (**Supplementary Table 1**). The *glyceraldehyde-3-phosphate dehydrogenase* gene (NCBI Reference Sequence: XM_006468885.2) of *C. sinensis* was utilized as an internal control, and the 2^-ΔΔCT^ method was employed to determine the relative expression profiles of the chosen genes (Livak and Schmittgen, 2001). The statistical significance of results from qRT-PCR analysis was assessed using an unpaired two-sided Student’s *t*-test through SPSS 25.0.

## 3 Results

### 3.1 Six BioProjects with 66 BioSamples were generated in this study

To better understand the disruptions in biological processes of *C. sinensis* caused by CLas infection, we carried out keyword searches within the SRA database in NCBI. A total of 6 BioProjects containing 66 BioSamples were collected and analyzed in this study. The 6 BioProjects included PRJNA951807 (Shahzad et al., 2023), PRJNA599503 (Peng et al., 2021), PRJNA579742 (Wu et al., 2020), PRJNA394061 (Zhao et al., 2019), PRJNA203307 (Martinelli et al., 2012, 2013), and PRJNA953196 (Liu et al., 2023) (**Table 1**) and detailed accessions of 66 BioSamples were listed in **Supplementary Table 2**. The 66 BioSamples included tissues from leaf, bark, root, fruit, and calyx abscission zones, divided into two groups: MOCK and HLB (**Table 1**).

**Table 1:** Details of RNA-seq data of *Citrus sinensis* associated with CLas infection used in this study.

Experiment	Group	Control group	Stress group	Comparison	Accession
Experiment 1	2018M11	Mild-2018M11-R1 Mild-2018M11-R2 Mild-2018M11-R3 Mild-2018M11-R4	Severe-2018M11-R1 Severe-2018M11-R2 Severe-2018M11-R3 Severe-2018M11-R4	C1	PRJNA951807
	2019M2	Mild-2019M2-R1 Mild-2019M2-R2 Mild-2019M2-R3 Mild-2019M2-R4	Severe-2019M2-R1 Severe-2019M2-R2 Severe-2019M2-R3 Severe-2019M2-R4	C2	
Experiment 2	Leaf	CK_L1 CK_L2 CK_L3	HLB_L1 HLB_L2 HLB_L3	C3	PRJNA599503
	Bark	CK_P1 CK_P2 CK_P3	HLB_P1 HLB_P2 HLB_P3	C4	
	Root	CK_R1 CK_R2 CK_R3	HLB_R1 HLB_R2 HLB_R3	C5	
Experiment 3	Leaf	CS-M_1	CS-HLB_1	C6	PRJNA579742
Experiment 4	Calyx Abscission zones	Rh1 Rh2	Rd1 Rd2	C7	PRJNA394061
		Dh1 Dh2	Dd1 Dd2	C8	
Experiment 5	Mature fruit	MF-CO	MF-SY	C9	PRJNA203307
		MF-CO	MF-AS	C10	
		MF-CO	MF-AH	C11	
	Immature fruit	IF-CO	IF-SY	C12	
		IF-CO	IF-AS	C13	
		IF-CO	IF-AH	C14	
	Mature leaves	ML-CO	ML-SY	C15	
		ML-CO	ML-AS	C16	
		ML-CO	ML-AH	C17	
	Young leaves	YL-CO	YL-SY	C18	
		YL-CO	YL-AS	C19	
		YL-CO	YL-AH	C20	
Experiment 6	Young leaves	MOCK1 MOCK2 MOCK3	HLB1 HLB2 HLB3	C21	PRJNA953196

### 3.2 WGCNA revealed key modules associated with CLas infection

**Figure 1:**
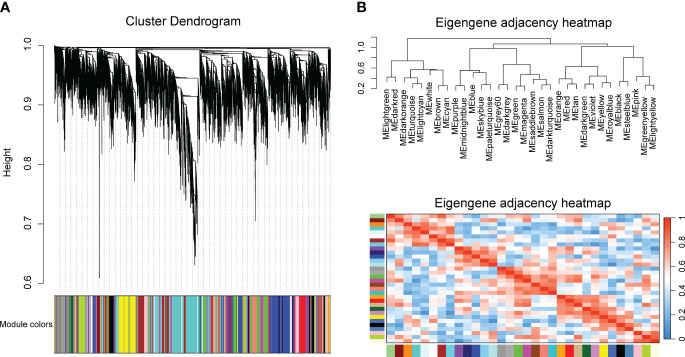
Clustering genes of *Citrus sinensis* into different modules base on the expression profiles of CLas infected samples by RNA-seq analysis. **(A)** Cluster dendrogram of all expressed genes. **(B)** Correlation heatmap of different modules.

**Figure 2:**
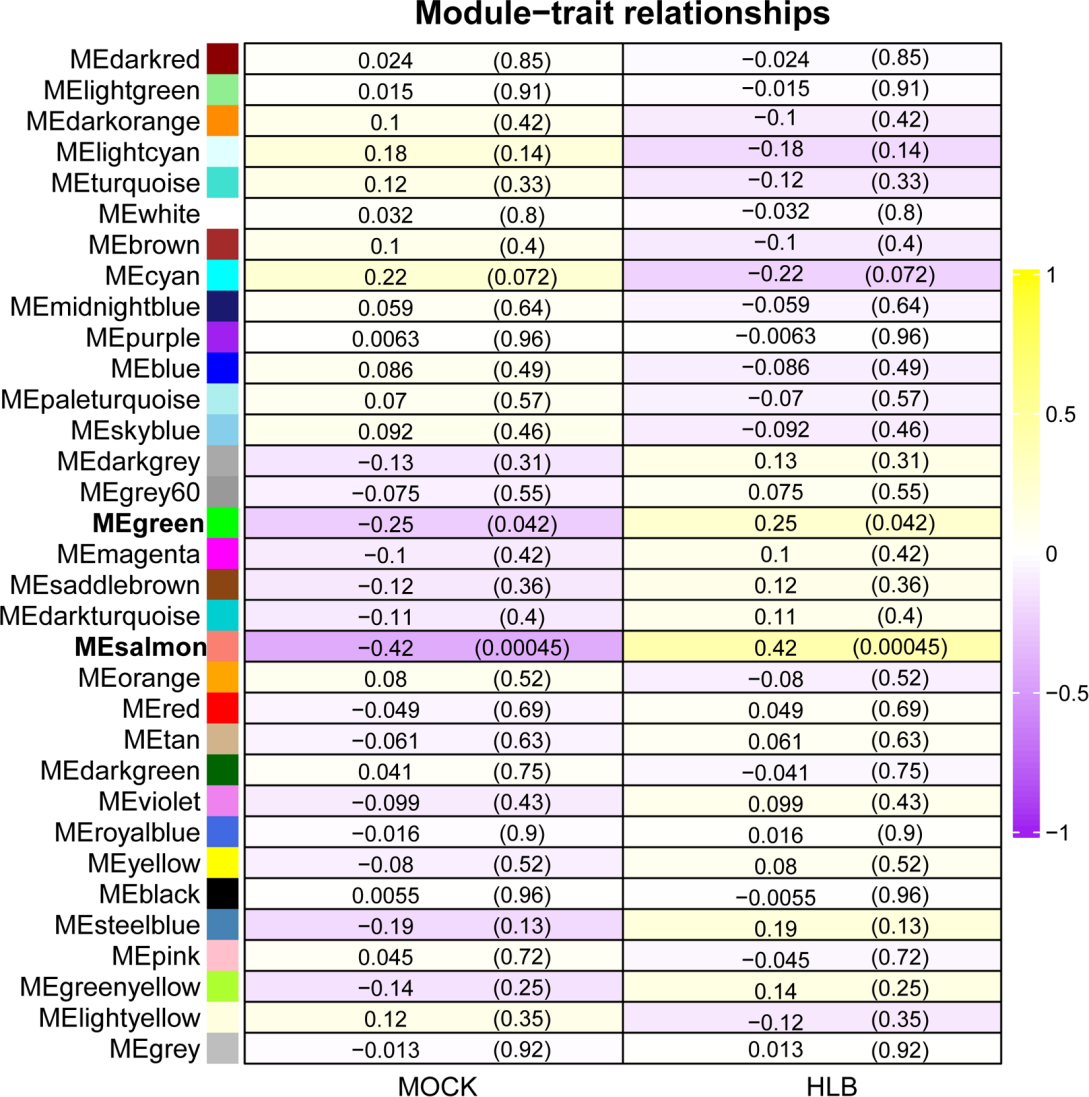
Identifying key modules of gene sets from *Citrus sinensis* associated with CLas infection by WGCNA. The numbers in the rectangular columns show the correlation coefficient and *P* value. The bold labels indicate significant modules.

To identify key modules related with CLas infection, we performed co-expression analysis with WGCNA. After normalizing and filtering the raw count values of all genes in *C. sinensis*, a total of 14,587 genes were retained. Due to the power-law distribution of the degree of nodes in a biological network, we calculated the soft threshold and determined that a power of 9 was appropriate for further analysis (**Supplementary Figure 1**). The remaining genes were clustered into 33 modules (**Figure 1A**) and the correlation between each module were calculated (**Figure 1B**). When conducting association analysis on the trait data of various BioSamples that were previously categorized as either ‘HLB’ or ‘MOCK’, two modules were identified as significantly associated with CLas infection. These modules were the salmon module and the green module (**Figure 2**). The salmon module showed a correlation of 0.42 with ‘HLB’ and a *P* value of 0.00045, while the green module had a correlation of 0.25 with ‘HLB’ and a *P* value of 0.042 (**Figure 2**).

### 3.3 Identification of candidate disease resistant and ROS scavenge gene networks through co-expression analysis

**Figure 3:**
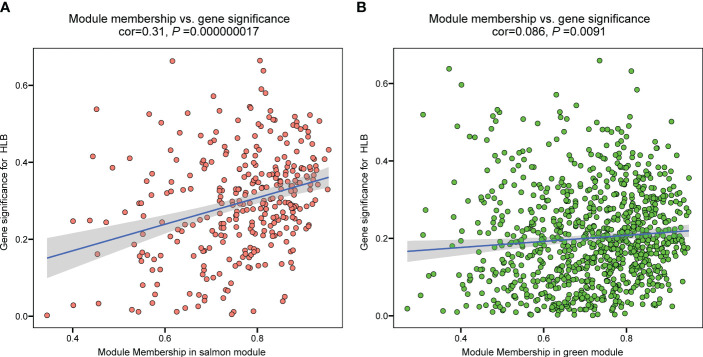
The relationship between module membership and gene significance of significant modules in the analysis of the RNA-seq data of *Citrus sinensis* associated with CLas infection. **(A)** Salmon module. **(B)** Green module.

**Figure 4:**
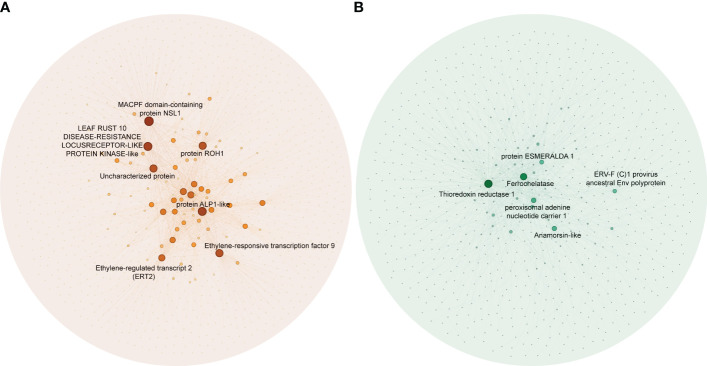
Co-expression networks in significant modules in the analysis of the RNA-seq data of *Citrus sinensis* associated with CLas infection. **(A)** Salmon module. **(B)** Green module. Blast analysis was employed to annotate the hub genes, using their descriptions found in the National Center for Biotechnology Information (NCBI) database.

To identify hub genes in the salmon and green module, we plotted gene significance (|GS|) versus module membership (|MM|) in coordinate system and genes with |MM| > 0.5 and |GS| > 0.5 were defined as hub genes (**Figure 3**). Then weight value of gene connectivity greater than 0.8^9^ were remained for co-expression network construction. In salmon module, 7 genes with top ranking were labeled with their gene functional descriptions. These genes included ‘MACPF domain-containing protein NSL1’ (*Cs_ont_2g004620*), ‘Leaf rust 10 disease-resistance locus receptor-like protein kinase-like’ (*Cs_ont_6g004280*), ‘protein ROH1’ (*Cs_ont_3g007360*), ‘Uncharacterized protein’ (*Cs_ont_7g000040*), ‘protein ALP1-like’ (*Cs_ont_4g011310*), ‘Ethylene-responsive transcription factor 9’ (*ERF9*) (*Cs_ont_2g022570*) and ‘Ethylene-regulated transcript 2’ (*Cs_ont_2g008210*) (**Figure 4A**; **Supplementary Table 3**). In green module, 6 genes including ‘Thioredoxin reductase 1’ (*TrxR1*) (*Cs_ont_1g029680*), ‘Ferrochelatase’ (*Cs_ont_4g005230*), ‘peroxisomal adenine nucleotide carrier 1’ (*Cs_ont_3g016200*), ‘protein ESMERALDA 1’ (*Cs_ont_2g012890*), ‘Anamorsin-like’ (*Cs_ont_7g018200*), and ‘ERV-F (C)1 provirus ancestral Env polyprotein’ (*Cs_ont_2g019740*) were identified (**Figure 4B**; **Supplementary Table 4**). These hub genes positive correlated with CLas infection would be key candidates for functional validation in further study.

### 3.4 DEGs varied in different experiments: a case study

**Figure 5:**
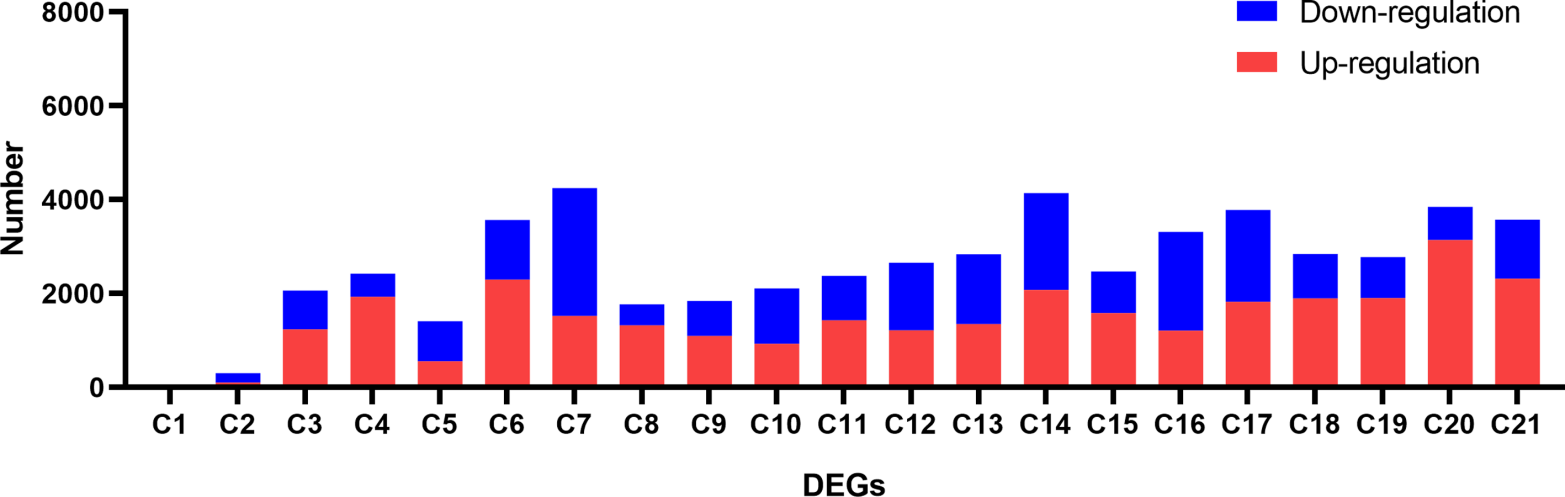
Identification of differential expressed genes (DEGs) between CLas infected and control samples in the analysis of the RNA-seq data of *Citrus sinensis*. The details for C1 to C21 are described in **Supplementary Table 6**.

**Figure 6:**
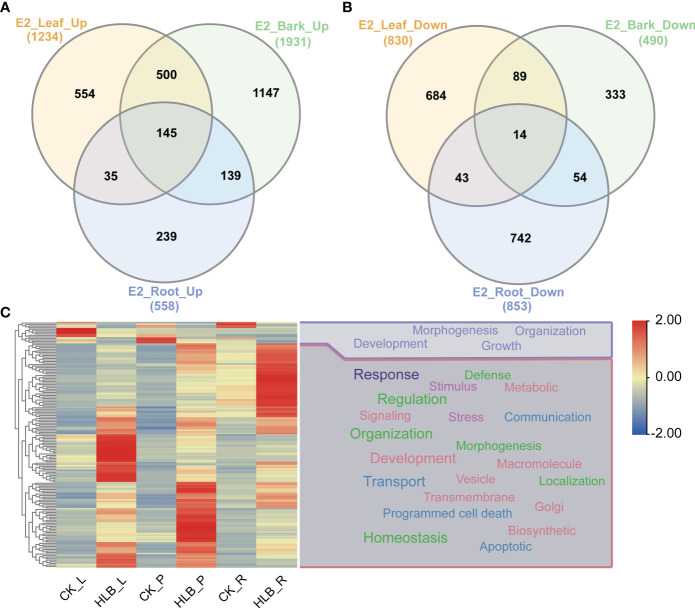
Differential expressed genes identification and GO enrichment analysis in experiment 2. **(A)** Common up-regulated genes. **(B)** Common down-regulated genes. **(C)** GO enrichment analysis. E2 signifies experiment 2, the specifics of which are documented in **Table 1**. The symbols L, P, and R denote leaf, bark, and root samples respectively.

We conducted DEGs identification to uncover the biological processes disrupted by CLas infection, resulting in the identification of diverse DEGs in different experiments (**Supplementary Tables 5**, **6**). A maximum of 4,250 DEGs were identified in C7, comprising 1,522 up-regulated genes and 2,728 down-regulated genes. In contrast, the minimum number of DEGs was observed in C1, where 5 genes were found to be down-regulated (**Figure 5**; **Supplementary Table 4**). Due to the limited number of DEGs in C1 and C2 from experiment 1, the subsequent analysis did not include these two groups of DEGs. Following that, we proceeded with a thorough analysis of experiment 2, which includes a variety of tissues, such as leaves, bark, and roots. The comparison between HLB and MOCK samples of leaves, bark, and roots resulted in the identification of 145 common up-regulated DEGs and 14 common down-regulated DEGs (**Figures 6A, B**). The analysis of GO annotation revealed that the commonly down-regulated DEGs are associated with ‘Development’, ‘Morphogenesis’, ‘Organization’, and ‘Growth’, while the up-regulated DEGs are associated with ‘Response’, ‘Regulation’, ‘Transport’, and ‘Homeostasis’, as well as other GO keywords like ‘Defense’, ‘Stimulus’, ‘Stress’ and ‘Programmed cell death’ (**Figure 6C**). In this study, we pay more attention to the GO enrichment results related with defense response, which were not detailed described in previous analysis (Peng et al., 2021).

### 3.5 PPI network analysis identified key SEO proteins

**Figure 7:**
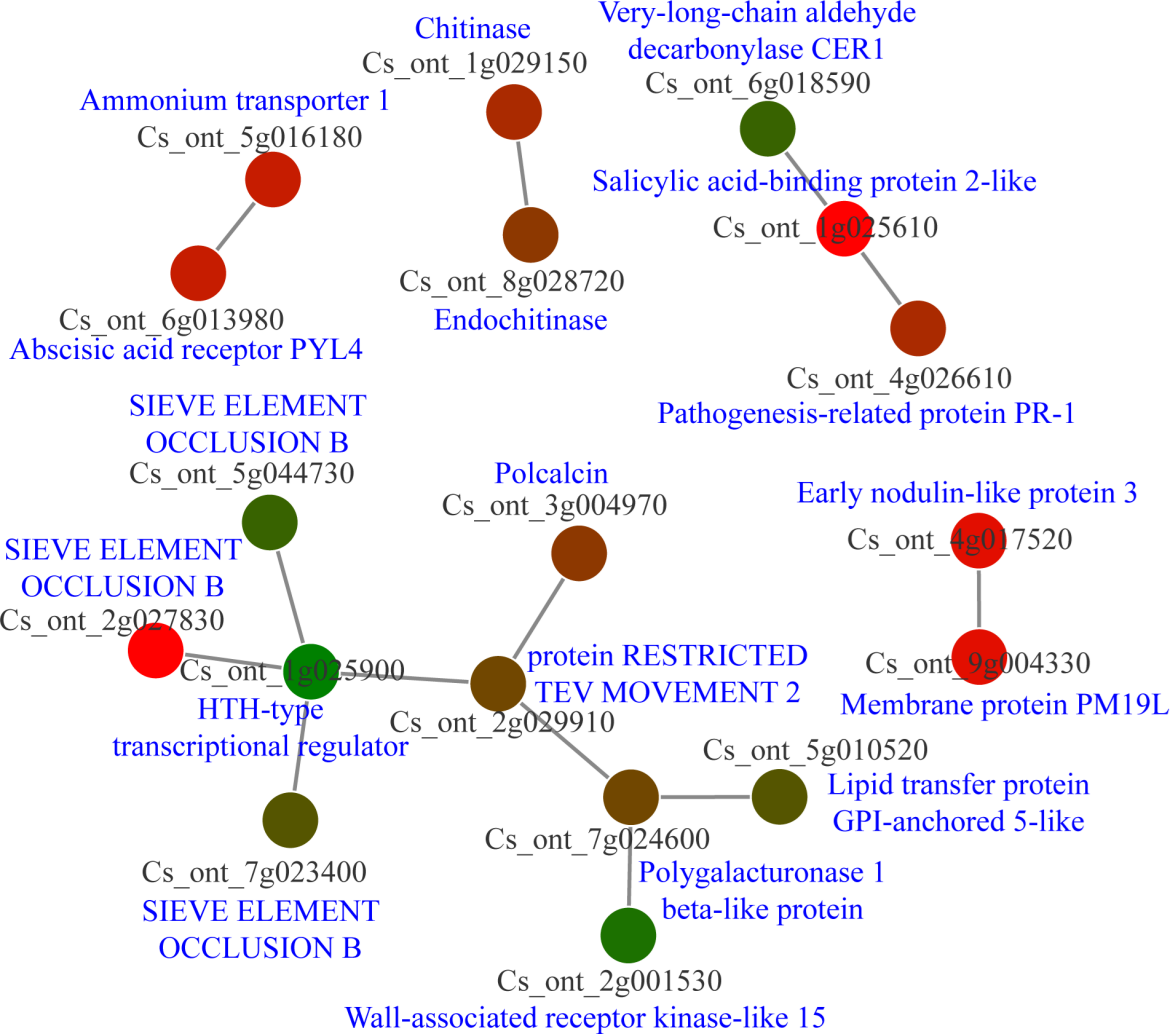
Protein-protein interaction (PPI) network analysis of core differential expressed genes in *Citrus sinensis* during CLas infection. PPI networks were derived using the STRING database, with each node arbitrarily assigned a hue from the rainbow spectrum. Detailed descriptions pertaining to each gene were sourced from the National Center for Biotechnology Information (NCBI) database.

The count function in Excel was used to obtain cDEGs, resulting in the generation of 114 cDEGs. Following this, the protein sequences of cDEGs were downloaded from the CPBD database and submitted to the String database for analysis of PPI networks. Five separate PPI networks were created (**Figure 7**; **Supplementary Table 7**). It is interesting to note that one PPI network consisted of nine nodes, with three of them being sieve element occlusion (SEO) B, one being an HTH type transcriptional regulator, and one being a wall-associated receptor kinase-like 15. Furthermore, there was another PPI network with three nodes, which consisted of very-long-chain aldehyde decarbonylase CER1, salicylic acid-binding protein 2-like, and pathogenesis-related protein PR-1. The other PPI networks also consisted of resistance associated genes like chitinase, abscisic acid receptor PYL4 (**Figure 7**). Our analysis indicated SEO proteins may be significantly involved in citrus-CLas interaction.

### 3.6 Defense responsive GO terms and secondary metabolic pathways were disturbed by CLas infection

**Figure 8:**
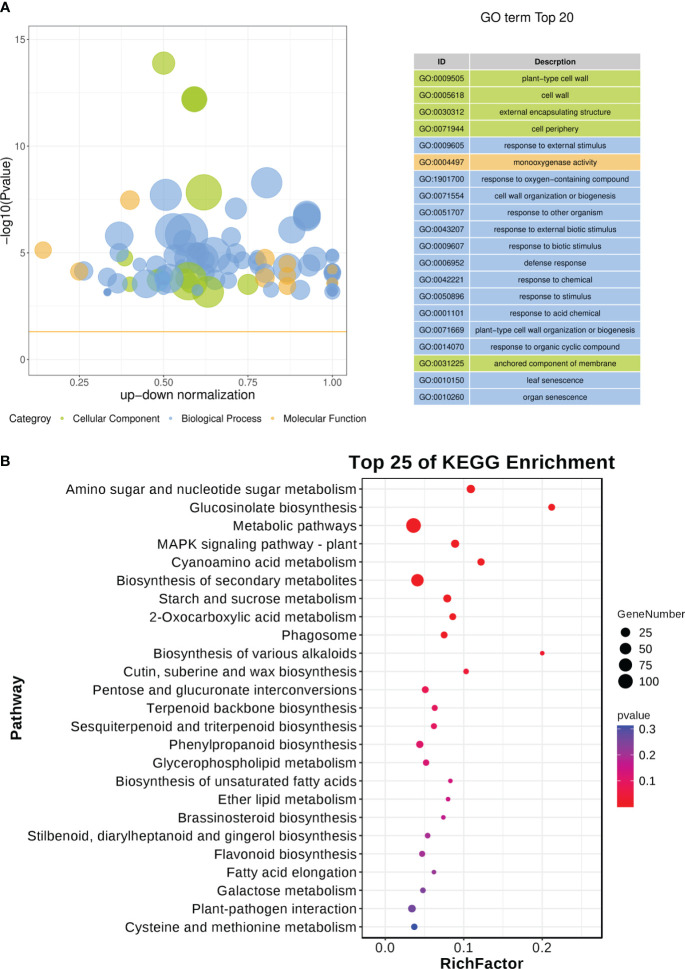
GO and KEGG pathway enrichment analysis of soft-core differential expressed genes in *Citrus sinensis* during CLas infection. **(A)** GO enrichment analysis of soft-core DEGs. **(B)** KEGG pathway enrichment analysis of soft-core DEGs. The enrichment GO terms and KEGG pathways were organized according to -log10 (*P* value), and the leading 20 enriched GO terms and the top 25 enriched KEGG pathways were subsequently visualized.

A total of 676 scDEGs were filtered and used for GO and KEGG pathway enrichment analysis. The GO enrichment analysis showed a significant influence on GO terms related to the cell wall, particularly GO:0009505 (plant-type cell wall) (**Figure 8A**; **Supplementary Table 8**). In addition, several defense related GO terms like GO:0009605 (response to external stimulus), GO:0009607 (response to biotic stimulus), and GO:0006952 (defense response) were identified (**Figure 8A**). Moreover, GO:0010150 (leaf senescence) was significantly enriched in this study (**Figure 8A**). KEGG pathway enrichment indicated various pathways were disturbed during CLas infection (**Figure 8B**; **Supplementary Table 9**). It is worth noting that sugar metabolism, including ‘Amino sugar and nucleotide sugar metabolism’, ‘Glucosinolate biosynthesis’, ‘Starch and sucrose metabolism’, and ‘pentose and glucuronate interconversions’, was affected (**Figure 8B**). Signaling pathways such as ‘MAPK signaling pathway-plant’ was altered (**Figure 8B**). Amount of biosynthesis pathways were impacted like ‘Cutin, suberine and wax biosynthesis’, ‘Terpenoid backbone biosynthesis’, ‘Sesquiterpenoid and triterpenoid biosynthesis’, ‘Phenylpropanoid biosynthesis’, ‘Brassinosteroid biosynthesis’, ‘Stilbenoid, diarylheptanoid and gingerol biosynthesis’, and ‘Flavonoid biosynthesis’ (**Figure 8B**). Additionally, there had been a change in the ‘Plant-pathogen interaction’ (**Figure 8B**). The expression of genes related to biotic stresses in *Citrus* spp. was found to be significantly influenced according to the GO and KEGG enrichment analysis.

### 3.7 Various enriched gene sets positively correlated with CLas infection

**Figure 9:**
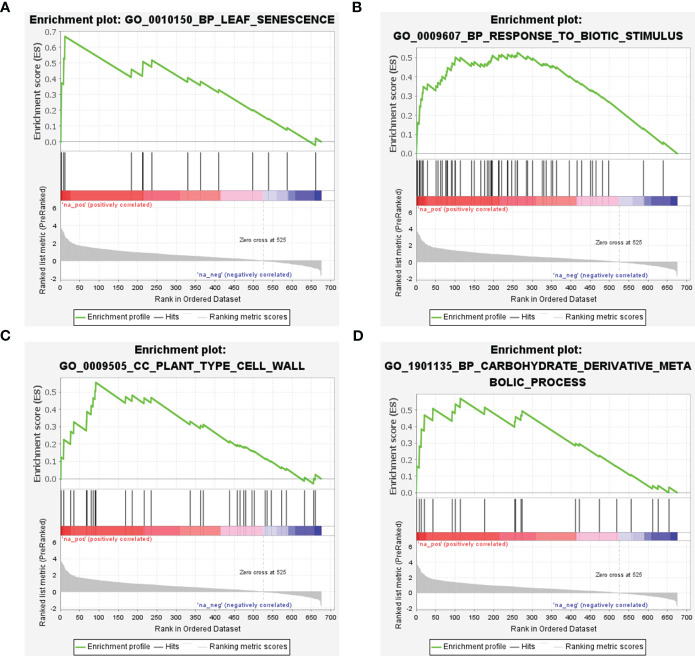
Gene set enrichment analysis of soft-core differential expressed genes in *Citrus sinensis* during CLas infection. **(A)** Leaf senescence. **(B)** Response to biotic stimulus. **(C)** Plant-type cell wall. **(D)** Carbohydrate derivative metabolic process. The enrichment score and the ranked list metric for each gene were individually ploted.

GSEA was conducted utilizing scDEGs to identify gene sets exhibiting positive correlations with CLas infection. The results of GSEA were in alignment with GO enrichment analysis (**Figure 9**; **Supplementary Table 10**). The GSEA revealed a significant number of up-regulated DEGs associated with GO:0010150 (leaf senescence), GO:0009607 (response to biotic stimulus), GO:0009505 (plant-type cell wall), and GO:1901135 (Carbohydrate derivative metabolic process) (**Figure 9**; **Supplementary Table 10**). These results indicated CLas infection interferes with multiple biological processes within citrus plants.

### 3.8 RNA-seq data validation by qRT-PCR analysis

**Figure 10:**
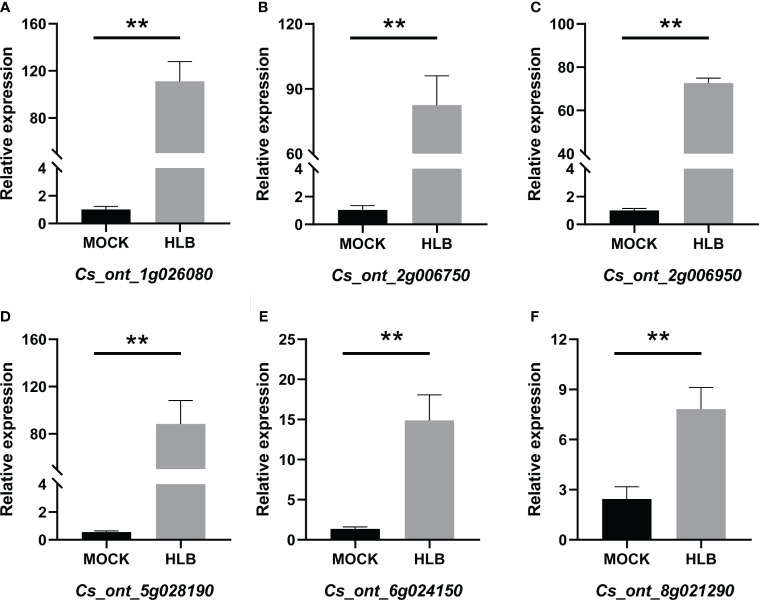
QRT-PCR analysis of the expression profiles of six candidate genes from *Citrus sinensis* during CLas infection. Relative expression of **(A)** *Cs_ont_1g026080*, **(B)** *Cs_ont_2g006750*, **(C)** *Cs_ont_2g006950*, **(D)** *Cs_ont_5g028190*, **(E)** *Cs_ont_6g024150*, and **(F)** *Cs_ont_8g021290*. The *C. sinensis GAPDH* gene was used as internal reference, three biological replicates were adopted, and the relative expression values were calculated by 2^-ΔΔCT^ method. The symbol ‘**’ denotes a *P* value of less than 0.01, indicating statistical significance.

QRT-PCR was employed to verify the accuracy of the RNA-seq data. Six DEGs that were found to be up-regulated in CLas-infected *C. sinensis* were randomly selected for further analysis, including vacuolar amino acid transporter YPQ1 (*Cs_ont_1g026080*), uncharacterized protein (*Cs_ont_2g006750*), glucose-6-phosphate/phosphate translocator 2 (*Cs_ont_2g006950*), kunitz trypsin inhibitor 3 (*Cs_ont_5g028190*), o-methyltransferase 3 (*Cs_ont_6g024150*), and glucose-1-phosphate adenylyl transferase large subunit 3 (*Cs_ont_8g021290*). The qRT-PCR analysis revealed an up-regulation of all chosen genes, consistent with the RNA-seq data (**Figure 10**). Therefore, this validation confirmed the reliability of the RNA-seq analysis.

## 4 Discussion

The citrus industry grapples with the destructive impact of HLB, a disease propagated globally causing significant damage (Wang, 2019; Hu et al., 2021). This study has sought to analyze the pathogenesis of CLas, despite the ambiguity surrounding its unculturable nature *in vitro*. In the realm of plant science, WGCNA has proven to be a powerful tool for exploring complex genetic traits and plant responses to various environmental factors (Mutinda et al., 2023). Unveiling these hub genes can lead us to the genetic controls underlying critical physiological traits, stress responses, and adaptation strategies in plants (Yu et al., 2023). Utilizing 289 transcriptome samples of *Dendrobium catenatum*, key genes including *DcCHIL*, *DcFLS*, *DcDFR*, and *DcWRKY3/4* were determined to react to methyl Jasmonate treatment. Further experimental data confirmed the role of *DcWRKY3/4* in regulating the metabolic pathway of flavonoids (Li et al., 2024). In the complex interplay between sorghum and the parasitic plant *Striga hermonthica*, the use of WGCNA facilitated the identification of several key genes playing a role in resistance response (Mutinda et al., 2023). During *Sporisorium scitamineum* infection, the transcriptomic profiling of sugarcane pinpointed 38 pivotal genes by WGCNA, incorporating those encoding chitinase, glutathione S-transferase, and heavy metal-associated isoprenylated plant protein (Wu et al., 2022). In this study, key gene modules were identified in *C. sinensis* during CLas infection. This was achieved through a co-expression analysis with WGCNA, retaining a total of 14,587 genes for analysis. Through our intricate research methodology, we identified two modules, namely ‘Salmon’ and ‘Green’, significantly associated with the infection. The hub genes identified within the ‘Salmon’ and ‘Green’ modules included *ERF9* (*Cs_ont_2g022570*), ‘Leaf rust 10 disease-resistance locus receptor-like protein kinase-like’ (*Cs_ont_6g004280*), and *TrxR1* (*Cs_ont_1g029680*). It has been demonstrated that *ERF9* is engaged in plant defense mechanisms against necrotic fungi (Maruyama et al., 2013). The leaf rust 10 disease-resistance locus receptor-like protein kinase-like protein is also implicated in the complex relationship between wheat and *Puccinia triticina*, which triggers leaf rust (Lee et al., 2020). *TrxR1* serves a crucial function in maintaining the equilibrium of redox reactions within plant cells (Gelhaye et al., 2005). The genes within these modules are involved in intricate processes such as disease resistance, reactive oxygen species (ROS) scavenging, and a range of other functional activities. Our study supplements these findings by identifying specific genes that might be involved in these complex processes.

Previous transcriptome analyses linked to HLB have pointed out significant influences on GO terms and KEGG pathways associated with stimulus and metabolic processes (Martinelli et al., 2012; Rawat et al., 2017; Arce-Leal et al., 2020; Liu et al., 2023). For example, during the asymptomatic stage of CLas infection, the most enriched biological process GO terms were associated with stimulus response, energy generation, lipid metabolism, and cellular homeostasis (Arce-Leal et al., 2020). In order to understand the biological processes affected by CLas infection, we performed GO enrichment analysis of DEGs. The identification of GO terms related to ‘Programmed cell death’ and ‘Homeostasis’ is noteworthy. Considering that HLB stimulates the accumulation of reactive oxygen species (ROS), triggers programmed cell death, and is perceived as an immune-mediated disease (Ma et al., 2022), Our findings further indicate that CLas infection significantly impacts the gene expression patterns related to programmed cell death and redox homeostasis. Furthermore, in the HLB-tolerant variety *Poncirus trifoliata*, there was a significant expansion in the number of genes associated with the MAPK signaling pathway (Bao et al., 2023). In contrast, the MAPK signaling pathway genes were significantly induced in another HLB-tolerant variety, *C. jambhiri*, but not in the HLB-susceptible variety *C. sinensis* (Yu et al., 2017). Nevertheless, our study revealed a slight activation of the MAPK signaling pathway following CLas infection, as determined by KEGG enrichment analysis. Moreover, our study identified activation in the metabolic pathway of brassinosteroid biosynthesis. Prior research has demonstrated that treatment with brassinosteroids notably mitigated the symptoms of CLas (Canales et al., 2016). Our findings further substantiate the understanding that citrus plants response to the CLas infection via conserved biological processes or metabolic pathways.

The PPI network analysis plays a critical role in illuminating the mechanics of transcriptional regulatory networks in plants (Alves et al., 2014; Konishi and Yanagisawa, 2019). The identification of 114 cDEGs and the subsequent PPI network analysis shed light on key responsive elements within the host. The PPI networks underscored the participation of numerous genes associated with resistance, accentuating the reaction of citrus plants to CLas infection. Noteworthy, *SEO* genes are responsible for the production of structural phloem proteins, which play an essential role in the wound-sealing process within the phloem (Ernst et al., 2012). The HTH-type transcriptional regulator has been observed to respond during instances of pathogen infection (Matoušek et al., 2015). The wall-associated receptor kinase serves as a crucial protein, facilitating resistance against pathogens in a variety of plants (Li et al., 2009; Qi et al., 2021a, Qi et al., 2021b). Another intriguing PPI network comprises very-long-chain aldehyde decarbonylase CER1, salicylic acid-binding protein 2-like, and pathogenesis-related protein PR-1. Salicylic acid-binding protein 2-like and pathogenesis-related protein PR-1 are integral components of the salicylic acid signaling pathway (Yang et al., 2022). However, the expression of CER1 was inhibited during salicylic acid treatment (Wang et al., 2021). The interplay of these proteins suggests a complex defense response, possibly involving pathways related to salicylic acid signaling and related defense pathways.

Furthermore, GSEA has emerged as a powerful method for interpreting gene expression data in the context of plant-pathogen interactions (Jiang et al., 2017). In rice-blast fungus interaction. multiple upregulated gene sets related to pathogen recognition, signal transduction, and the production of antimicrobial compounds in the rice cultivars resistant to the blast fungus were identified using GSEA (Wei et al., 2013). An analysis of the transcriptomic response of Flax to *Fusarium oxysporum* revealed a notable enrichment of genes associated with the terpenoid backbone biosynthesis pathway by GSEA (Galindo-González and Deyholos, 2016). Additionally, our GSEA results validated the conclusions obtained from the GO enrichment analysis. Many up-regulated DEGs were involved in leaf senescence, response to biotic stimulus, plant-type cell wall, and carbohydrate derivative metabolic process, strengthening the evidence of the extensive impact of CLas infection at the cellular and molecular levels. There has been a demonstrated correlation between leaf senescence and the susceptibility of plants to pathogens (Jarosch et al., 2005; Häffner et al., 2015). Notably, GO:0010150 (leaf senescence) was significantly enriched, supporting observations of premature aging in HLB-infected citrus trees. In this study, results of enrichment analysis revealing a substantial impact on GO terms related to the cell wall and various defense responses. These variations in the host’s transcriptome profile underscore the pervasive and disruptive nature of HLB disease, affecting both structural and defense-related components of the host.

## 5 Conclusion

In summary, a comprehensive analysis of *C. sinensis* RNA-seq data in relation to CLas infection was conducted in this study. Hub genes positive correlated with CLas infection, including ‘Leaf rust 10 disease-resistance locus receptor-like protein kinase-like’, *ERF9* and *TrxR1*, were identified using WGCNA. Moreover, GO and KEGG enrichment revealed various biological processes and metabolic pathways, including ‘Programmed cell death’, ‘Homeostasis’, ‘MAPK signaling pathway’, and ‘Brassinosteroid biosynthesis’ were significantly influenced. PPI network analysis identified key responsive proteins such as SEO, HTH type transcriptional regulator, and wall-associated receptor kinase-like 15. Furthermore, GESA indicated a significant number of up-regulated DEGs associated with leaf senescence, biotic stimulus, plant-type cell wall and carbohydrate derivative metabolic process. These findings would broaden our understanding of citrus-CLas interactions and pave the way for future studies on the intricate transcriptional regulations in *C. sinensis* during CLas infection.

## Data availability statement

The datasets presented in this study can be found in online repositories. The names of the repository/repositories and accession number(s) can be found in the article/**Supplementary Material**.

## Author contributions

RL: Funding acquisition, Investigation, Methodology, Software, Writing – original draft, Writing – review & editing. XW: Investigation, Software, Writing – original draft. YH: Methodology, Writing – original draft. GH: Conceptualization, Funding acquisition, Project administration, Writing – original draft, Writing – review & editing.

## Supplementary material

Supplementary Figure 1 The scatter and bar diagrams for **(A)** scale independence, **(B)** mean connectivity, **(C)** soft connectivity, and **(D)** check scale free topology generated during progress of WGCNA.
