# Supplementary figures and images for: Analysis of huanglongbing-associated RNA-seq data reveals disturbances in biological processes within Citrus spp. triggered by Candidatus Liberibacter asiaticus infection
Source: Front Plant Sci. 2024 Apr 10;15:1388163. doi: 10.3389/fpls.2024.1388163 (PMC11039969; doi:10.3389/fpls.2024.1388163)

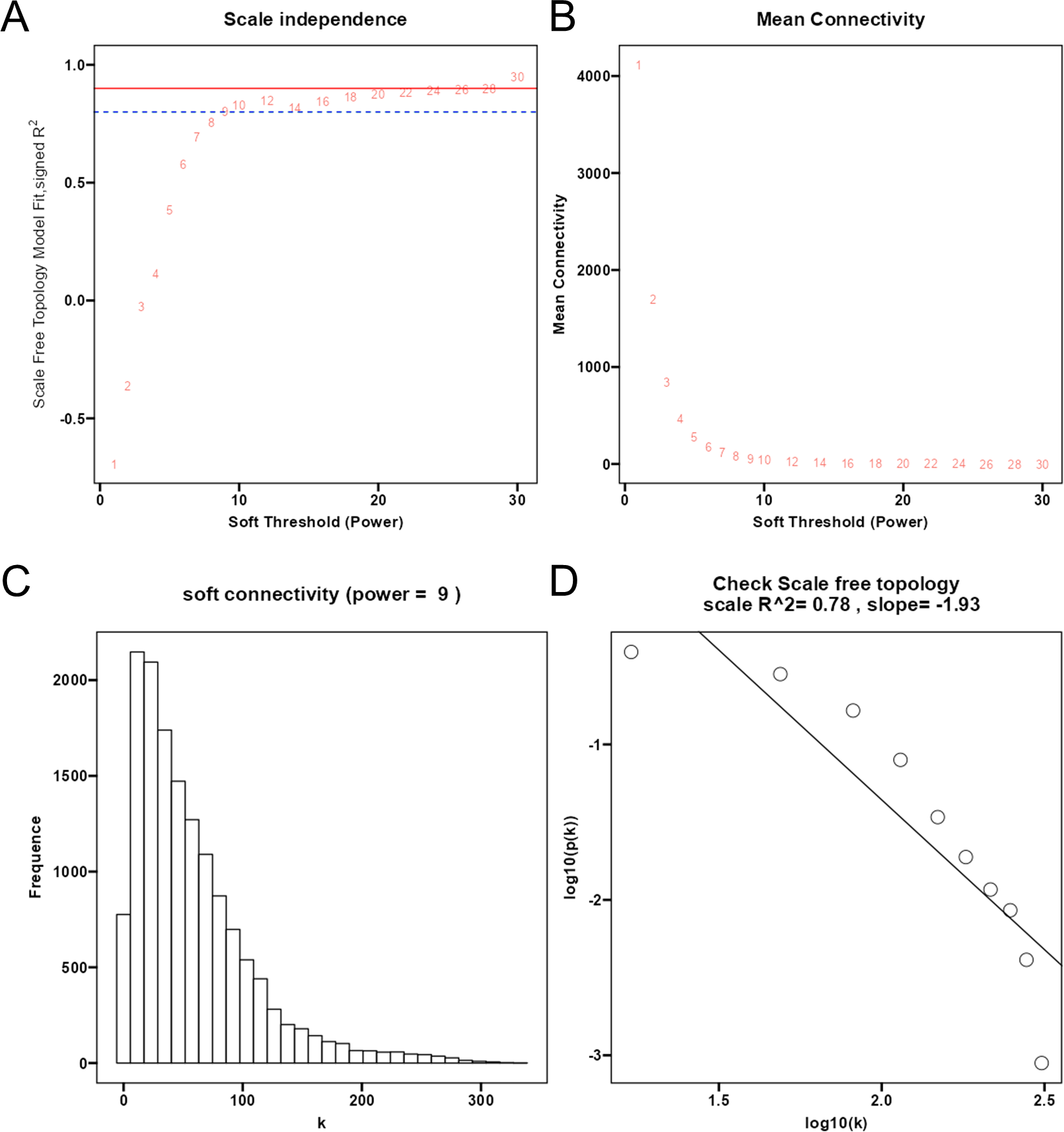

Supplement: Supplementary Figure 1 — The scatter and bar diagrams for (A) scale independence, (B) mean connectivity, (C) soft connectivity, and (D) check scale free topology generated during progress of WGCNA. [file Image_1.tif]
